# Evaluation of Negative Pressure Wound Therapy dressing in the management of mommy makeover surgery wounds

**DOI:** 10.1080/23320885.2025.2450102

**Published:** 2025-01-09

**Authors:** Matilde Tettamanzi, Federico Ziani, Anna Manconi, Giovanni Arrica, Claudia Trignano, Edoardo Filigheddu, Silvia Rampazzo, Ilaria Ginatempo, Michail Sorotos, Fabio Santanelli di Pompeo, Corrado Rubino, Emilio Trignano

**Affiliations:** a Plastic Surgery Unit, University Hospital Trust of Sassari, Sassari, Italy; b Department of Medicine, Surgery and Pharmacy, University of Sassari, Sassari, Italy; c Department of Biomedical Sciences, University of Sassari, Sassari, Italy; d Department of Neuroscience, Mental Health, and Sense Organs, Faculty of Medicine and Psychology, Sapienza University of Rome, Rome, Italy; e Sant’ Andrea Hospital, Rome, Italy

**Keywords:** Mommy makeover, body contouring, wound healing, dressings, negative wound pressure therapy

## Abstract

**Background:**

This investigation explores the potential impact of Negative Pressure Wound Therapy (NPWT) dressing on mommy makeover surgical wounds. The focus is on optimizing the healing process and post-surgical care to mitigate complications like wound dehiscence, seroma, and hematoma.

**Patients and methods:**

A prospective study spanned from October 2015 to April 2022, involving 40 patients undergoing mommy makeover surgery for aesthetic purposes. The randomized division resulted in two groups. Group one (*n* = 20) had donor sites covered with NPWT dressing, while group two (*n* = 20) received standard dressings lacking known healing-promoting agents. The assessment of complications served as an index of NPWT efficacy, and scars were evaluated using the Vancouver Scale.

**Results:**

Immediate post-surgical use of NPWT dressings significantly expedited wound healing compared to fine-mesh gauze dressings. Furthermore, it almost eradicated discomfort and pain in all patients, indicating excellent compliance. Patients tolerated NPWT well, with no instances of dressing failure or non-compliance.

**Conclusion:**

This study underscores the utility of NPWT dressing in managing mommy makeover surgery wounds. The dressing’s bio-occlusive properties create an optimal environment for wound healing, simultaneously minimizing pain, discomfort, and preventing key complications such as seroma and unfavorable scar appearance.

## Introduction

Mommy makeover surgery is a popular cosmetic surgery technique that has gained significant attention in recent years and is one of the most frequently performed procedures in aesthetic plastic surgery. Combining abdominoplasty and breast surgery has become a popular choice for many patients. The trend of combining these procedures initially arose from performing abdominoplasty alongside intraabdominal or gynecologic surgery [1]. Initially, the focus was on ensuring safety and minimizing localized complications, such as issues with wound healing. As surgeons broadened their approach to encompass abdominoplasty alongside more distant procedures like breast surgery, it became evident that these individual procedures have minimal adverse impacts on each other and remain unaffected when performed in combination [2,3]. The techniques and technologies have continuously improved to decrease complications and to enhance the overall results, especially in mammoplasties and breast reconstructions [4–6] The primary objective of our work is to elucidate the efficacy of NPWT Dressing after mommy makeover surgery in order to encompass expeditious postoperative recovery, fast and good wound healing with whole low complication rate and minimal need for postoperative antalgic therapy. The prevention of complications and good scar appearance represents one the main goal in aesthetic plastic surgery, related to the high expectations of the patients and the elective nature of this kind of surgery. The care of clean, close surgical incisions varies from preoperative prophylactic measures and microbial sealants to intraoperative devices like prophylactic gentamycin-collagen sponges, extending to postoperative interventions. Postoperative measures encompass traditional dressing with sterile dry gauzes, debriding agents, and topical antimicrobial dressing, as well as more advanced wound dressings designed to promote the proliferative phase of wound healing. These advanced dressings include hydrocolloids, the topical application of autologous blood products, growth factors, cultured skin, and Negative Pressure Wound Therapy (NPWT) [7,8]. To date, extensive evidence exists, which demonstrate the benefits of negative pressure dressing in the treatment of open wounds, however its application in close incisions is not well characterized and described. Our study takes part of a small but growing number of clinical studies based on the hypothesis that negative pressure dressing improves healing of close and sutured wounds. The main objectives of our study include gaining insights into the functioning of NPWT and assessing its effectiveness in reducing the incidence of complications such as infection, dehiscence, seroma, hematoma, skin and fat necrosis, as well as issues like skin and fascial dehiscence or blistering. Additionally, we aim to examine other variables affected by the application of NPWT, such as re-operation and re-hospitalization rates, time to dry wound, and potential cost savings.

## Materials and methods

**Figure 1.**
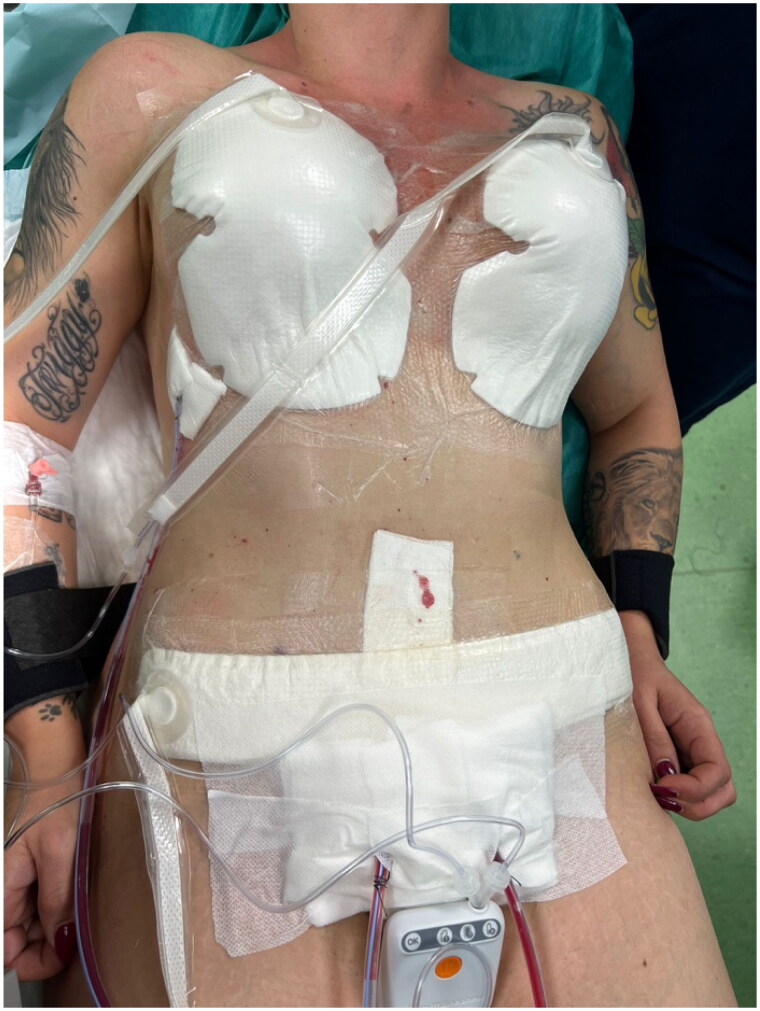
The donor sites are covered using NPWT dressing.

This study evaluated patients’ medical records who underwent mommy makeover surgery between 2015 and 2022. Every patient’s indication for surgery was an aesthetic correction aimed to improve the appearance of the abdomen by removing excess skin and fat and tightening the underlying muscles, to repair herniation of the abdominal wall and to correct diastasis of the rectus abdominis muscles, and to improve the appearance of the breasts, and the sagging or drooping. It aims to enhance breast firmness, reposition the nipple-areola complex, and create a more aesthetically pleasing breast shape, enhancing overall breast contour, symmetry, and appearance. The patients were fully informed about the surgery, indications, and possible complications (i.e. bleeding, infection, and scarring) and consciously consented to the surgery. Patients underwent a thorough medical evaluation to be considered eligible for the procedure. Exclusion criteria comprised the presence of local or clinical signs of infection, diseases affecting the healing of sutured areas (e.g. diabetes, neuropathies, and immunological disorders like systemic lupus erythematosus), and the use of systemic corticosteroids or immunosuppressants. The study enrolled all patients without exclusion criteria who provided informed consent. Patient unawareness of the applied dressing was maintained, and they were randomly allocated into two groups. The study group consisted of forty females with a mean age of 52.3 years (range 34–69 years). To ensure comparable characteristics, factors such as age, systemic diseases, and others were considered during group allocation. The patients were randomized into two groups, and in the first group of 20 patients (Table 1), NPWT dressing was used to cover the donor sites. (Figure 1). The NPWT system (PICO device, Smith and Nephew Medical Ltd, Hull, UK) has been employed on an outpatient basis in the plastic surgery department in recent years. This device ensures a continuous negative pressure of −80 mmHg, falling within the evidence-based therapeutic range. Its dressings are constructed with four layers, facilitating the delivery of NPWT and predominantly removing wound exudate through evaporative loss. These dressings can be worn for up to 10 days. The silicone wound contact layer prevents granulation tissue from growing into the dressing material, contributing to patient comfort during dressing changes and supporting the re-epithelization process. Patients can safely be discharged with the NPWT dressing in place, as there are no specific contraindications for its use. The treatment duration was determined by the surgeons based on specific criteria: discontinuation of NPWT was guided by the appearance of the wound—defined as “healed” when achieving a Vancouver Scar Scale (VSS) score of 2 or below across relevant parameters—and a reduction in exudate. In the second group of 20 patients, donor sites were closed intra-operatively with a standard dressing lacking any known healing-promoting agents. To assess the comfort experienced by patients, they were asked to respond to questions regarding confidence and comfort during daily activities. Additionally, they provided feedback on pain quality, and the Vancouver Scar Assessment Scale was employed to evaluate scar healing. This scale (Table 3) incorporates four variables: vascularity, height (thickness), pliability, and pigmentation, each with four to six possible scores. The total score ranges from 0 to 14, where 0 indicates normal skin. According to the Vancouver Scar Assessment Scale criteria, we define "healed" as achieving a score of 2 or below in the scale parameters. This benchmark allows us to objectively assess the point at which healing is considered complete. The number of complications served as an index to evaluate the efficacy of NPWT dressing compared to the standard dressing.

**Table 1.** Patient characteristics by treatment group and incision Area.

Characteristic	NPWT (Abdomen)	Control (Abdomen)	NPWT (Breast)	Control (Breast)
Number of Patients	20	20	20	20
Mean Age (years)	52.3 ± 8.9	52.3 ± 9.2	52.3 ± 8.8	52.3 ± 9.1
BMI (kg/m²)	26.1 ± 3.2	25.8 ± 3.5	24.5 ± 3.0	25.2 ± 3.1
Smoking Status (n, %)	3 (15%)	4 (20%)	2 (10%)	3 (15%)
Diabetes (n, %)	1 (5%)	1 (5%)	0 (0%)	1 (5%)

**Table 3.** The Vancouver Scale.

THE VANCOUVER SCALE			
Vascularity	Pigmentation	Pliability	Height
0 = Normal	0 = Normal	0 = Normal	0 = Flat
1 = Pink	1 = Hypopigmentation	1 = Supple	1 = <2 mm
2 = Red	2 = Mixed	2 = Yielding	2 = 2-5 mm
3 = Purple	3 = Hyperpigmentation	3 = Firm	3 = >5 mm
		4 = Ropes	
		5 = Contracture	

## Results

Dressings in both patient groups remained in place for a minimum of 10 days and a maximum of 22 days, with a range of 12–22 days. The median length of treatment until healing was 16.25 days (standard deviation = 9.5; range: 7–31). The follow-up period ranged from a minimum of 12 weeks to a maximum of 22 months. The number of NPWT dressing packs per patient varied from 1 to 7 (note: 7 NPWT dressing packs consist of 14 dressings). This implies that, despite clinic visits, a new NPWT dressing pack was not always opened, given that the supplied 2 AA lithium batteries lasted up to 7 days. No reports of pain or discomfort associated with continuously high pressure from NPWT were documented. On the contrary, NPWT alleviated patient anxiety and reduced the pain and discomfort associated with frequent dressing changes. All donor site wounds healed and required no additional dressing by postoperative day 19. In the second group of patients treated with the standard dressing, five patients experienced itch from day 3, four patients until day 5, and two patients until day 7. The remaining patients experienced itch only on the day of onset.

## Scale results

**Figure 2.**
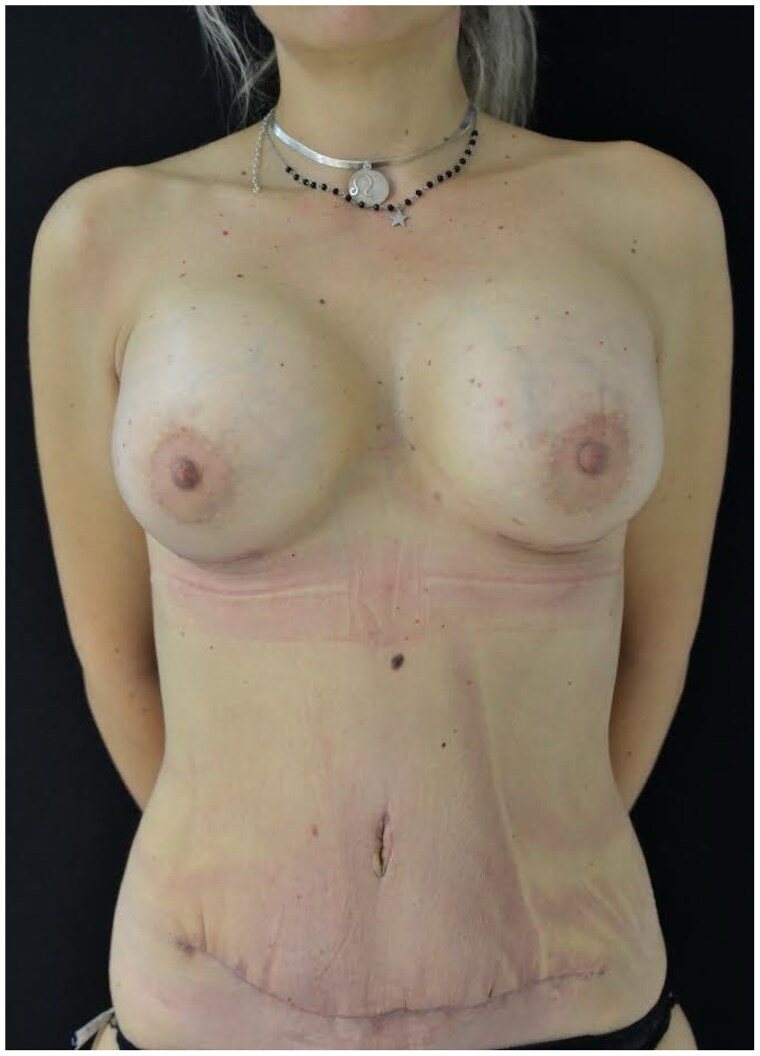
Postoperative view after 1 month. In this patient the donor sites were covered using the NPWT dressing.

**Figure 3.**
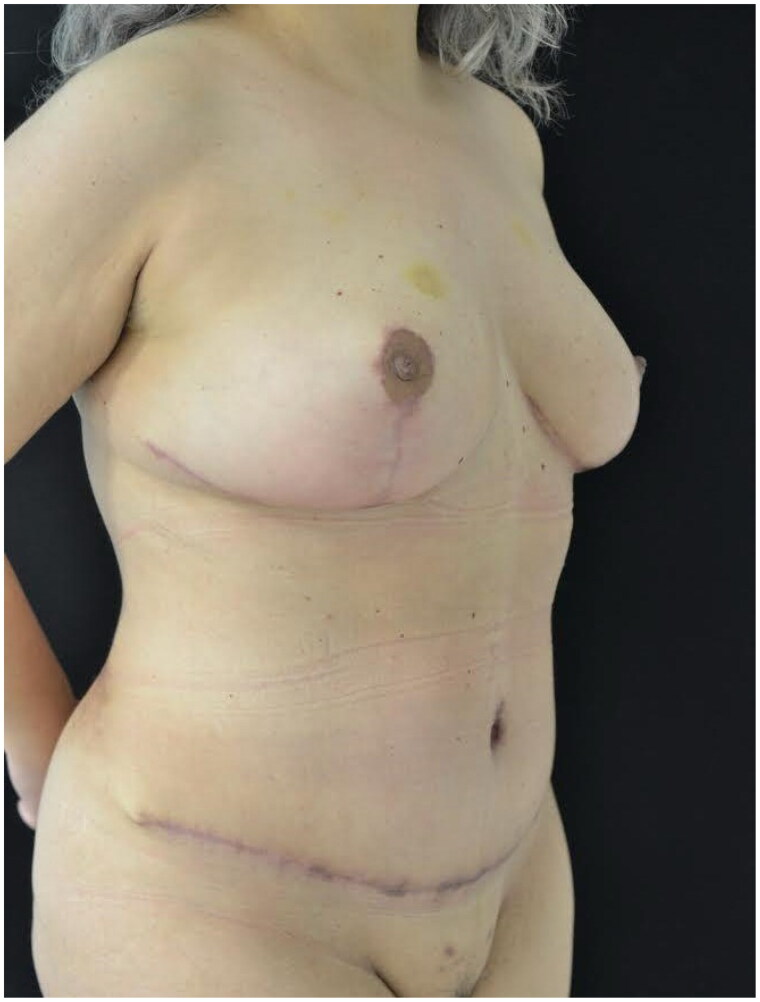
Postoperative view after 1 month. In this patient the donor sites were covered using the NPWT dressing.

All scar areas were assessed by the physicians during the study. At 12-weeks follow-up, difference was detected in the resulting scar and healing time among the patients under evaluation (Table 2). The Vancouver Scale [9] (Table 3) consists of four variables: vascularity, height (thickness), pliability, and pigmentation. Each variable has four to six possible scores. A total score ranges from 0 to 14, whereby a score of 0 reflects normal skin. Fourteen patients reported a score of 0, five patients reported a score of 1 and only one patient scored 2 in the group treated with the NPWT dressing (Figure 2). In the other cohort treated with the standard dressing (Figure 3), eight patients scored 0, seven patients reported a score of 1, three patients scored 2 and two patients marked 2 on the scale. All patients tolerated the NPWT well, with no instances of dressing failure or non-compliance, and no reported pain during dressing changes (Tables 4 and 5). Our study demonstrated the NPWT system’s capability to manage clinically relevant fluid volumes under both high and low exudate conditions. It illustrated how the NPWT dressing effectively removes wound fluid through absorption and subsequent evaporation facilitated by a high-moisture vapor transmission rate upper film. No instances of infection or bleeding requiring further treatment were observed in either group. At the 1-year follow-up, doctors detected no discernible difference in resulting scar appearance or healing time among the evaluated patients.

**Table 2.** Healing time and complications by treatment group and incision Area.

Outcome	NPWT (Abdomen)	Control (Abdomen)	NPWT (Breast)	Control (Breast)
Median Healing Time (days)	16.25 (7–31)	22.1 (10–35)	16.25 (7–31)	22.1 (10–35)
Pruritus (*n*, %)	1 (5%)	5 (25%)	1 (5%)	5 (25%)
Seroma (*n*, %)	0 (0%)	2 (10%)	0 (0%)	0 (0%)
Wound Dehiscence (*n*, %)	0 (0%)	1 (10%)	0 (0%)	1 (10%)
Other Complications (*n*, %)	0 (0%)	0 (0%)	0 (0%)	0 (0%)

**Table 4.** Vancouver Scar Scale scores at 12 weeks by treatment group and incision area.

Vancouver Scar Scale Score	NPWT (Abdomen)	Control (Abdomen)	NPWT (Breast)	Control (Breast)
Score 0	14	8	14	8
Score 1	5	7	5	7
Score 2	1	3	1	3
Score 3 or higher	0	2	0	2
Mean VSS Score	0.9 ± 0.6	1.3 ± 0.8	0.8 ± 0.5	1.5 ± 0.9

**Table 5.** Patient-reported comfort and pain levels by treatment group and incision Area.

Parameter	NPWT (Abdomen)	Control (Abdomen)	NPWT (Breast)	Control (Breast)
Average Pain Score (VAS, 0–10)	2.3 ± 1.1	3.8 ± 1.5	1.9 ± 0.9	3.5 ± 1.3
Dressing Changes (average)	1	3	1	3
Patient-Reported Comfort (*n*, %)	15 (75%) comfortable	10 (50%) comfortable	17 (85%) comfortable	15 (75%) comfortable

## Discussion

Mommy makeover, a widely sought-after cosmetic surgery procedure, has garnered considerable attention in recent years. It not only enhances overall life satisfaction and contentment with one’s health and physical appearance but also contributes to increased emotional stability [10,11]. Notably, individuals with depressive tendencies have exhibited significant improvement following aesthetic abdominoplasty and mastopexy, suggesting that its impact extends beyond mere aesthetics to encompass the broader physical well-being of patients [12–14].

Concerns surrounding the safety of mommy makeover surgery have been prevalent among both patients and surgeons. However, numerous studies have consistently demonstrated that, when performed by experienced and qualified surgeons, this procedure is associated with low complication rates [15]. Strategic patient selection and optimization are crucial factors for maximizing positive outcomes and preventing complications. Furthermore, consideration of individual patient factors, such as smoking, obesity, and pre-existing medical conditions, is essential as they can influence the risk profile and overall success of the procedure [16,17].

According to published case series, local complications are more prevalent than those with systemic repercussions following mommy makeover procedure. Approximately 10–20% of patients experience local complications, while fewer than 1% suffer from systemic complications [18]. The significance of proper patient selection and thorough preoperative assessment cannot be overstated in minimizing the risk of complications. Despite the generally favorable outcomes associated with abdominoplasty combined with breast surgery, it is imperative to acknowledge potential complications [19].

Risk factors for assessing post-operative complications in aesthetic plastic surgery can be categorized as surgery-related (incision placement, surgical site contamination, technique, operative time, estimated blood loss) or patient-related (morbid obesity, comorbidities, drugs, nicotine abuse) [7]. Aesthetic patients typically have a low rate of serious comorbidities but are demanding in terms of results [20].

Superficial wound healing issues and minor wound dehiscence are frequently reported complications in mommy makeover procedures, and in particular with the abdominoplasty surgery, often attributed to incision tension [11,21]. Our study aimed to validate the feasibility and effectiveness of NPWT dressing in managing post-abdominoplasty and post-mastopexy wounds and preventing post-surgical complications.

No dressing can be deemed universally ‘ideal’ since existing dressings exhibit distinct properties [22]. The emphasis is on dressings that create an optimal setting for moist wound healing and proficient exudate management, all while alleviating pain and discomfort. The single-use negative pressure wound therapy dressing has revolutionized our wound management promoting patient well-being and compliance. We recognize that variability in Vancouver Scale scores across the follow-up period may be influenced by differences in healing stages, as well as the diverse anatomical locations and incision types included in this study. To manage these sources of variability, we used stratified randomization to ensure balanced distribution of patient characteristics between the NPWT and control groups and considered the different incisions as summarized in Table 2. In addition, VSS scores were analyzed at 3 months as illustrates Table 4, to account for the impact of time on scar maturation. By categorizing follow-up intervals and grouping incisions by anatomical location in our analysis, we aimed to reduce variability and improve the comparability of healing outcomes. Future studies with larger sample sizes could further stratify by incision type and timing, allowing for more precise insights into NPWT’s effects on specific wound types and healing phases.

In summary, our study suggests that applying NPWT dressing to surgically closed wounds enhances healing characteristics compared to ordinary gauze dressings, particularly in cases involving wound tension, extensive soft tissue trauma, excessive drainage, or a risk of subdermal hematoma formation. It also leads to a significant reduction in exudate formation, enabling early drain removal and decreasing hospitalization duration, thereby reducing costs. NPWT proves to be a cost-effective intervention, with cost savings from a shortened hospital stay and prevention of post-operative surgical site infections offsetting the initial intervention cost [23].

While our study demonstrates the efficacy of Negative Pressure Wound Therapy (NPWT) dressings in enhancing wound healing after mommy makeover surgery, certain limitations should be noted. Firstly, this study included a relatively small cohort of 40 patients, which may limit the generalizability of the findings. A larger sample could provide more robust data on NPWT efficacy and a more comprehensive understanding of its impact across diverse patient populations. The prospective design and randomized assignment aimed to reduce bias; however, the study was conducted at a single institution, which may affect the applicability of the results to broader clinical settings. Multi-center trials would be beneficial for validating these findings. Moreover, our assessment of wound healing primarily relied on the Vancouver Scar Scale and patient-reported outcomes. While these measures provide valuable insights into healing quality, additional objective measurements, such as biochemical markers of wound healing, could offer a more comprehensive evaluation of NPWT’s effects. These limitations underscore the need for future research involving larger, multi-center studies to fully substantiate the role of NPWT in aesthetic surgery wound management. Despite these constraints, our findings suggest a positive impact of NPWT dressings on postoperative healing and patient comfort and endorse the approach of using NPWT as a prophylactic measure over clean, closed surgical incisions immediately after surgery. This evidence holds great importance in the management of wounds in aesthetic surgery, catering to high-demand patients and ensuring a swift recovery with optimal wound healing and scar appearance.

## Conclusion

Mommy makeover surgery remains a popular and effective surgical procedure for enhancing abdominal and breast aesthetics and elevating patient satisfaction, serving as a potent body contouring technique. Our findings suggest that NPWT may optimize healing in postoperative incisions by potentially shortening the time to achieve wound healing benchmarks. Analysis of Vancouver Scar Scale scores indicates that NPWT-treated incisions tend to reach improved healing states earlier compared to control. The application of the NPWT device demonstrates notable effectiveness, leading to a quicker and higher-quality healing process and a subsequent decrease in the overall complication rate. Further research with larger samples could help to more conclusively quantify these time-dependent changes in healing.

## Data Availability

Authors should declare where the data supporting their findings can be found. Data can be deposited into data repositories or published as supplementary information in the journal. This article does not contain any studies with animals performed by any of the authors.
